# In Memory

**Published:** 1883-07

**Authors:** F. Peabody, W. H. Morgan, A. Wilkes Smith, J. F. Canine

**Affiliations:** Committee; Committee; Committee; Committee


### In Memory.

The following action was taken at the recent meeting of the Kentucky State Dental Society held in Louisville, Kentucky:

Mr. President:—While we welcome, with pleasure, our professional brothers from far and near, at this our yearly reunion, still we look in vain for the one familiar face that every member of our association was ever glad to see, and whose greeting and welcome to each and every one, was so frank, so hearty, that all felt it was earnest and true.

Our brother and friend Dr. Wm. H. Goddard can meet with us no more in this world. But on the other side, when we cross the dark river, we cannot but feel that the same earnest, warm greeting will be extended to us from our friend, who has gone before.

No member of the profession was more eager after the discovery of any new light that would aid us in making the highest attainments.

Ever at our State Association when his health would permit; always from his first membership in prompt attendance on the yearly meeting of the American Dental Association; at the time of his death occupying its highest official position, we feel that we as an Association, and that the American Association, can never too fully appreciate his personal and professional worth.

As an Association, we feel that this slight tribute to his worth is but a poor expression of our appreciation of his real merit, but we desire that a copy of this action be sent to his family, with whom we deeply sympathize, and that a copy be forwarded the Dental Journals.

F. Peabody,

W. H. Morgan,

A. Wilkes Smith,

J. F. Canine,

Committee.

